# From scribbling to painting: children’s drawing as an evolutionary laboratory for multisensory and sensorimotor integration

**DOI:** 10.3389/fnhum.2026.1876364

**Published:** 2026-08-04

**Authors:** Monica Gori, Giulio Sandini

**Affiliations:** 1 Institute for Human and Machine Cognition (IHMC), Pensacola, FL, United States; 2 UVIP-IIT, Istituto Italiano di Tecnologia, Genova, Italy; 3 RBCS-IIT, Istituto Italiano di Tecnologia, Genova, Italy

**Keywords:** children’s drawing, cognitive neuroscience, embodied development, multisensory integration, painting, sensorimotor training

## Abstract

Adult painting emerges from a sophisticated multisensory integration between vision, touch, proprioception, and motor control, taking shape as a form of active Bayesian inference about the external environment. The evolutionary origins (and survival value) of such embodied coordination, however, remain poorly explored. Traditional literature views children’s drawing as a passive output of perceptual-motor-cognitive development. This work proposes the opposite hypothesis: that drawing acts as an active form of natural training, serving as an evolutionary laboratory for sensorimotor calibration. Through a parallel analysis between graphic stages and neuroscientific milestones, we propose that each stroke provides persistent and informative feedback. The kinematic parameters of the gesture (velocity, curvature, pressure) are linked to stable visuo-tactile outcomes on the page, surpassing the effectiveness of other traceless motor experiences. Bayesian modeling suggests that drawing may involve iterative active experiments in which predictions about gestural outcomes are generated, compared with observed feedback, and used to update generative models that align internal and external sensory maps. This ecological process could help to learn eye-hand coordination and to update the formation of spatial-bodily representations in a period of life characterized by a fast physical growth of the body parts and maturation of the motor plant. By redefining drawing as developmental training in perceptual and creative growth, the hypothesis opens new perspectives on early multisensory plasticity.

## Introduction

Adult painting represents the mature expression of a cognitive process deeply rooted in the multisensory integration of vision, touch, proprioception, and movement. At the same time, painting retains a profoundly ludic dimension, a playful engagement that fuels intrinsic motivation and creative exploration. As recently highlighted, the act of painting is not merely the application of color onto a surface, but rather a dynamic dialog between mental intention, bodily gesture, and multiple sensory feedbacks (Gori and Sandini, 2025). However, the evolutionary origins and survival value of this sophisticated embodied coordination remain poorly understood. One possible hypothesis is that scribbling may have emerged, at least in part, as a pre-verbal communicative act, allowing meaning to be expressed through gesture, trace, and visual symbolism before the full development of spoken language. In this sense, scribbling, often described as abstract or undifferentiated, may represent an initial form of graphic action that precedes and scaffolds more structured representations, including writing. Traditional perspectives tend to interpret children’s drawing, from early scribbles to more realistic depictions, primarily as an outcome of perceptual, motor, and cognitive development. Here, we adopt a complementary view: drawing activities, including scribbling, free drawing, and even coloring predefined shapes, may also function as active drivers of development by providing structured opportunities for sensorimotor coupling and feedback. Through a theoretical analysis of graphic development stages in relation to neuroscientific findings, we explore how graphic action may serve as a natural laboratory for sensorimotor calibration. Each stroke generates persistent traces that link kinematic properties of movement (e.g., velocity, curvature, pressure) to stable visual and tactile consequences, potentially supporting the alignment of sensory and motor representations. By redefining drawing as a possible multisensory training ground, this contribution invites a reconsideration of perceptual development as an intrinsically creative and graphic process.

## Multisensory art and movement

Artistic practices have long been regarded as a privileged window onto perception and cognition, yet most empirical work has focused on adult observers and on vision alone. In a recent contribution, Gori and Sandini (2025) emphasized that painting can be understood as the outcome of a creative intentional process deeply rooted in the integration of multisensory perception and motor action. The painterly act emerges from the desire to transform, through color and bodily movement, an absent image into something visible and tangible, where gesture constitutes the point of contact between sensory experience, mental content, and esthetic outcome. An activity which exercise not only sensorimotor coordination but, more interesting in a cognitive sense, the ability to anticipate the consequences of goal directed actions. From this perspective, vision is not the only relevant channel: the use of a brush, the fingers, or other tools jointly engages tactile, proprioceptive, and motor systems, generating an intrinsically multisensory painting experience as well as the affordance of drawing tools and techniques. As also discussed by Calvert et al. (2004), the interaction among sensory channels provides a privileged window into how the brain constructs the meaning of perceptual experience, beyond a purely visual analysis of the image. In animals indeed converging influences from visual, auditory, and somatosensory cortices onto output neurons of the superior colliculus (Stein et al., 1993; Wallace et al., 1993). According to multisensory integration models proposed by Ernst and Banks (2002), the brain combines information from multiple senses in a statistically optimal way, improving perceptual precision, accuracy, and reaction times (Ernst and Bulthoff, 2004). We have shown that the interaction between visual and tactile signals can facilitate motion perception, suggesting a tight dialog between different sensory channels even in dynamic perceptual tasks (Gori et al., 2011). In painting, this principle means that the surface and the pictorial material are not only seen, but also anticipated and experienced tactually and proprioceptively through the body’s contact with the tool and the support. Action and perception are therefore intrinsically connected, and the artist does not simply “see” what is represented, but simulates and plans the painterly gesture, translating mental images into action programs that incorporate sensory and motor parameters.

Within this framework, the finished artwork is only the visible trace of a much richer perceptual-motor-cognitive process, much of which remains implicit in the space of action. From the movement standpoint, the painterly gesture is not a merely technical means for applying color to a surface, but a fundamental component of the expressive content of the work. Variations in force, speed, trajectory, and movement amplitude translate into strokes, textures, and forms that convey emotional states, intentions, and meanings, in line with the tradition linking movement kinematics and graphic form. Research on body schema and motor planning further shows that biomechanical constraints and regularities in arm trajectories systematically shape the resulting graphic mark: painting is therefore also a product of the body’s limits and possibilities. The relationship between stroke curvature and speed, the temporal modulation of action, and eye-hand coordination all contribute to defining the painter’s style and the motor legibility of the artwork for the observer. In short, pictorial art emerges from a dynamic interplay among multisensoriality, movement, and creativity. Scribbling is therefore not only a visual product, but an embodied process in which perception, action, and mental representation co-determine one another throughout creation and fruition.

This theoretical framework, which unites multisensory processing and movement in the adult artist, offers a fertile basis for asking how these processes emerge and develop during childhood, when children begin to explore the world and represent it through drawing.

## Multisensory development in childhood

As discussed in the previous section, the link between art and multisensoriality highlights painting as a privileged context for observing the integration of vision, touch, proprioception, and movement. The pictorial experience does not involve only the visual analysis of the finished image: during the creative act, the artist must coordinate information from the surface and the tool, through tactile and haptic feedback, from the position and movement of the body through proprioception, and from the visual content being generated, thereby constructing a coherent multisensory representation of both gesture and outcome. In this sense, painting also relies on cognitive operations that integrate the anticipated visual result with the motor commands needed to achieve it. This forward-looking planning links perception, action, and goal-directed intention. Models of multisensory integration, as described above, suggest that the brain learns to combine different sensory sources in a statistically advantageous way, improving perceptual precision and motor control, and scribbling, drawing and painting represents an emblematic example of this cooperation among the senses in the generation of graphic marks. In doing so it provides the system with the tools needed to anticipate how to transform an imagined graphic result into actions.

From a developmental perspective, however, numerous studies show that the ability to integrate multiple sensory cues optimally is not present from early infancy, but tends to emerge later, often only in late childhood. Gori et al. (2008) showed that children aged 5–6 years and until 8 years do not effectively combine visual and haptic information about object shape, often relying on a single sense rather than integrating the available signals. Similarly, Nardini et al. (2008) reported that optimal multisensory cue integration for spatial localization, for example between vision and audition, begins around age 8 and is completed by around ages 12–14, with younger children tending to use only one sensory cue. Petrini et al. (2014) extended these findings to the auditory-haptic domain, showing that up to 10–12 years of age children do not integrate haptic and auditory signals well when estimating size, reaching adult-like benefits only later in development. Research on the influence of early sensory experience, including cases of congenital or late visual deprivation, further confirms that the quality and timing of multisensory experiences shape the organization of body-space maps, with substantial delays in integration in late-blind individuals (Gori et al., 2026). These findings suggest that the sophisticated interaction between multisensoriality and movement that characterizes adult painting cannot be taken for granted in children, but must be understood within a system under construction, in which different sensory and motor channels mature at different rates and require experience, exploration, and practice to become effectively coordinated. Childhood drawing can therefore be viewed not only as a graphic product, but also as a window onto the developmental state of multisensory mechanisms: the ways in which children use the body, explore the surface, coordinate vision and hand, and “feel” their own gesture reveal how far they have progressed along the path toward adult-like sensory integration. Furthermore, the predictive ability required to transform a mental simulation of a graphic sign into a sensorimotor act may provide an additional measure of cognitive development.

## Art and motor development in childhood

If multisensory development is still not fully defined during the earliest period of life, motor development is likewise a gradual and highly structured process. As discussed above, the link between drawing and movement highlights that the graphic gesture is not a mere technical means, but the very core of expressive activity, in which the kinematic and dynamic properties of movement determine the form, style, and emotional content of the mark. During the painterly act, variations in pressure, speed, trajectory, and movement amplitude generate unique strokes, textures, and structures that carry the imprint of the acting body, translating mental intentions into visible traces through a continuous dialog between motor planning and sensory feedback. In child development, these dynamics emerge through progressive stages that reflect the acquisition of fine motor control, eye–hand coordination, and gesture planning. These stages are not merely expressive, but also index the development of fundamental sensory, motor and cognitive abilities: from proximal control involving shoulder and arm to distal control involving fingers and wrist, from gross motor activity to fine motor refinement, and eventually to gesture programming that anticipates the outcome. Delays in these phases, such as excessively large strokes beyond the age of 3 years or difficulty copying shapes, may indicate motor immaturity or problems in perceptual-motor integration, underscoring the importance of drawing as a diagnostic and rehabilitative tool in psychomotor and possibly cognitive development. In parallel with what is observed in adult painting, childhood drawing reveals how movement evolves from chaotic exploration to intentional expression, shaped by bodily constraints and practical experience. In this sense, the child’s graphic activity is not only a product of development, but also a privileged observation point for understanding how motor control, visual guidance, and body representation gradually come together.

## The emergence of drawing

Scientific literature describes the development of children’s drawing as a continuous, evolutionary process shaped by the interaction of fine motor skills, eye-hand coordination, perceptual awareness, and representational intention, with well-defined stages first outlined by pioneers such as Luquet (1927) and Kellogg (1969) and later confirmed by empirical work and later enriched by empirical work (Quaglia et al., 2015). The progression of children’s drawing thereby mirrors the maturation of motor, perceptual, cognitive and multisensory capacities (Long et al., 2024; Toomela, 2003), in line with research on the development of visuo-tactile and multisensory integration. In the uncontrolled scribbling phase (approximately 12–24 months), the child produces random marks on the page, using a palmar grip and large, global movements of the arm and shoulder, with predominantly proximal control and still-gross motor patterns (Longobardi et al., 2015). At this stage, multisensory development is characterized by a unisensory dominance (e.g., for shape touch/proprioception over vision) and immature integration between sensory channels, consistent with findings showing that visuo-haptic integration is not yet optimal in early childhood (Gori et al., 2008). From a perceptual and sensorimotor standpoint, drawing appears as a causal exploration of the gesture-mark relationship, with limited eye–hand coordination yet a basic sensitivity to visuo-tactile correspondences, in line with evidence of early visuo-tactile colocalization in the first year of life.

In the controlled scribbling phase (2–3 years), more structured graphic forms emerge, such as simple vertical lines and circles, and the child begins to adopt a more refined tripod grip. Motorically, this stage reflects a shift toward more distal control (elbow, wrist, and gradually fingers) and a greater degree of intentional, goal-directed planning of the graphic gesture (Quaglia et al., 2015). In parallel, multisensory development begins to show early signs of cross-modal facilitation: vision increasingly guides touch in spatial calibration. Perceptually and sensorimotor-wise, awareness of one’s own gesture increases, reaching actions become more visually guided, and a first calibration of sensory frames begins. During the preschematic phase (4–7 years), the child represents human figures and objects in a symbolic and often chaotic way, typical of the “tadpole” figure and other stylized forms. Fine finger motor control consolidates, graphic actions follow more organized gestural sequences, and increasing lateralization becomes evident. Multisensory integration, however, is still not optimal until around 6–8 years of age; multiple studies show that children continue to rely predominantly on a single sense, with statistically optimal multisensory integration emerging only later, closer to late childhood. Perceptual–sensorimotor development during this phase remains markedly egocentric, with immature spatiotemporal integration and a basic body schema, consistent with work documenting the gradual maturation of temporal and spatial integration of multisensory information.

In the schematic phase (7–9 years), the child produces more standardized and recognizable graphic schemes (for example, the canonical “house” and increasingly detailed human figures), often anchored to a baseline that structures the space of the page. Motorically, gesture planning capabilities emerge, and movements become more proportional, including at the emotional-expressive level of the drawing. Research indicates that around the age of 8 years, multisensory integration approaches statistical optimality, yielding measurable advantages when vision and touch are combined relative to the use of a single modality. In parallel, spatial representation becomes more stable, eye-hand coordination becomes more advanced, and body awareness becomes more integrated, consistent with work describing the development of multisensory space perception and the progressive alignment between body maps and external space. Finally, in the realistic phase (from about 9 years onward), drawings show the acquisition of complex graphic concepts such as perspective, depth, and occlusion, with greater attention to spatial relations and visual details. Fine motor control is now highly precise, enabling self-correction and continuous monitoring of the gesture, features typical of a mature motor system. From a multisensory perspective, children reach an “adult-like” profile, with flexible integration and the ability to synchronize information from sensors that may be potentially asynchronous in time, in line with studies on the maturation of multisensory integration during adolescence. Perceptually and sensorimotor-wise, a more explicit visual realism emerges, supported by calibrated body maps and an implicit motor simulation that underlies both action and the understanding of others’ actions. The previous section thus outline a deep interweaving between stages of children’s drawing and the maturation of perceptual, motor, and multisensory processes, with the graphic gesture functioning as a “mirror” of development. It has been suggested that the control of the stroke refines eye-hand coordination and gestural planning (Lowenfeld, 1947). Furthermore, the production of symbols consolidates mental categories and communicative intentionality; drawing, conceived as “externalized thought,” precedes and supports spoken language and stimulates executive functions (Kellogg, 1969). Neuroscience studies link these phases to cortical plasticity and experience-dependent learning, with delays sometimes signaling impairments such as dysgraphia. In this context, drawing remains a unique diagnostic tool for tracing integrated development across perception, action, and representation.

## Mechanisms of multisensory training in childhood drawing

**Figure 1:**
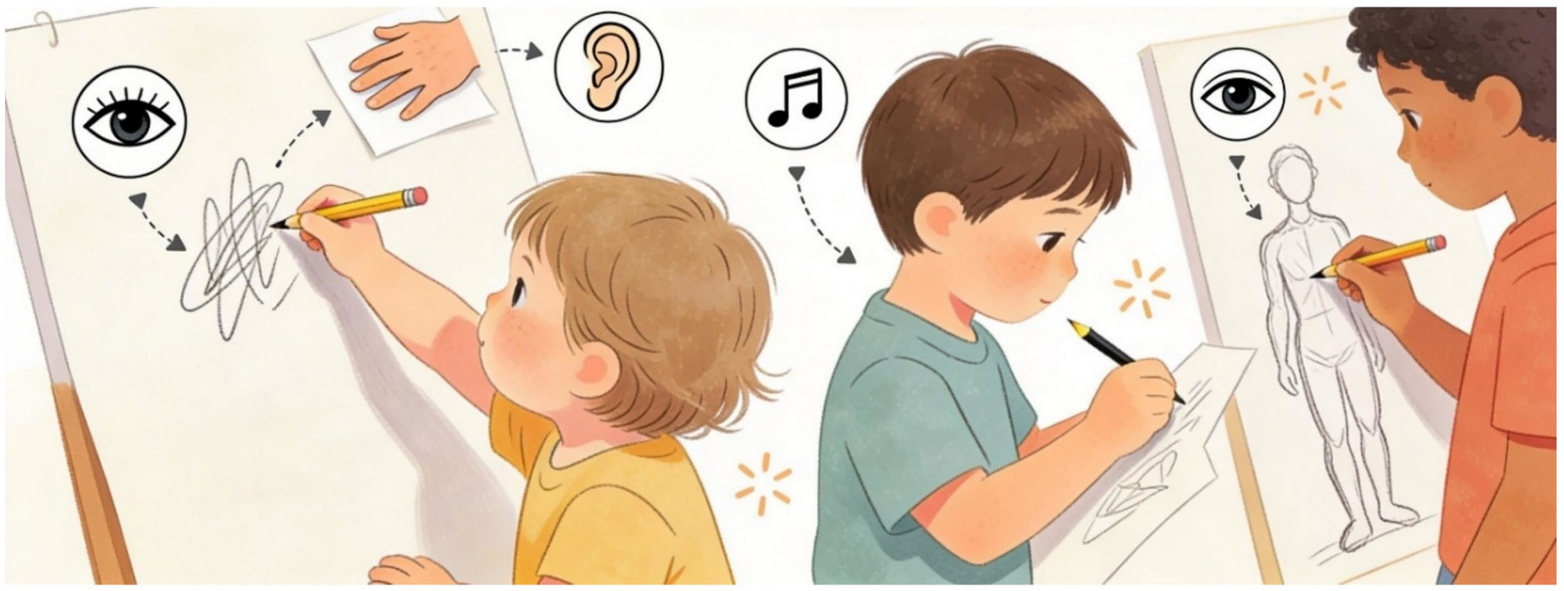
Image illustrating the association between multisensory processing and sensorimotor integration in childhood drawing. The figure illustrates how visual, tactile, proprioceptive, and auditory inputs might jointly contribute to the development of drawing actions across childhood. In the earliest phase, children engage in exploratory scribbling, with large proximal movements, while the hand, paper, and stroke sounds provide immediate sensorimotor feedback that supports basic gesture-outcome associations. In this stage, probably calibration process is active. At older ages, drawing becomes progressively more controlled: eye-hand coordination improves, movements become more distal and intentional, and children learn more effectively the consequences of their own body movements and start integrating sensory information. In later stages, more accurate multisensory integration and refined motor planning support schematic and realistic drawing, with vision providing richer spatial representations that helps children translate internal representations into structured graphic output and multisensory integration occurs.

So far, we have seen that there is a tight parallel between children’s drawing and the development of their motor, perceptual, and multisensory abilities, suggesting that drawing mostly reflects emerging competences. But what if the relationship were reversed, and drawing was instead an active training system for these very components? Drawing simultaneously engages multiple sensory channels, creating an ecologically rich learning environment and functioning as a form of natural, self-organized practice. Consider, for example, the visuo-tactile-proprioceptive association: while drawing, the child’s hand feels the resistance of the paper and the flow of the color (haptic feedback), while the eyes track the emerging trace. This visuo-tactile colocalization may be used to refine body and spatial maps, compensating for early immaturities in visuo-tactile development, as suggested by Gori et al. (2008). For the auditory component, the sound of the contact with the surface and the sound of the chalk on the surface or the scratching of the pencil provides temporal cues that synchronize gesture and perception, prefiguring the adult pattern of audio-haptic integration (Petrini et al., 2014). Training through drawing could thus foster the development of these circuits, reinforcing the binding of action and its sensory consequences in time. From an evolutionary perspective, drawing may share some of its advantages with other exploratory and noisy activities, such as playing with sand or striking objects together, like flintknapping, where early humans (and children in experimental studies) shaped stones by percussive actions, making the action-sensory consequence relation explicit. This continuity with more basic forms of sensorimotor interaction is consistent with developmental work on multisensory calibration, which emphasizes the role of experience in building integration across sensory channels, as seen in studies where vision and touch calibrate each other through repeated exploratory actions Finally, from a sensorimotor perspective, each stroke is an intentional action followed by immediate feedback (the gesture-trace contingency), implementing a simple but powerful loop of motor prediction and implicit simulation. It is precisely this sensorimotor association that we propose as the key mechanism through which drawing may function as a training device in the child: repeated, intentional actions generate robust forward models, which are then refined by the continuous comparison between predicted and observed outcomes (Figure 1). In the next section we will describe the theoretical framework underlying this mechanism, framing drawing as active sensorimotor experimentation and as a form of ecological training for the construction of an internal model supporting mental simulation and the execution of intentional gestures.

## The theory of sensorimotor association in development

**Figure 2:**
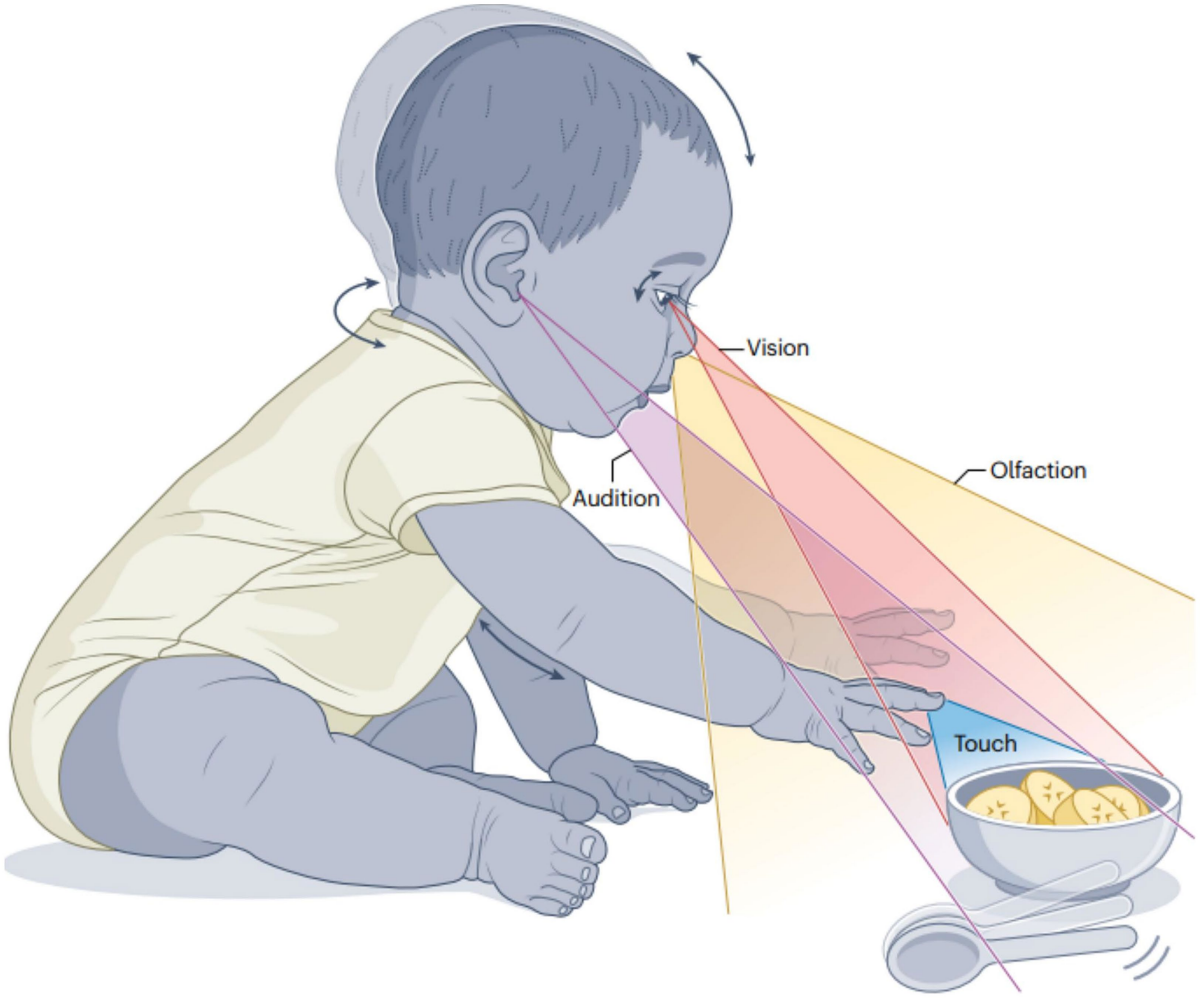
Spatial information is subject to different filters and distortions as it approaches the different senses. Tactile spatial locations are presented directly to the skin surface. Olfactory and auditory spatial signals are less precise than visual spatial signals. These spatial cues are coded within different frames of reference and change as the body changes posture (black arrows). From Gori et al. (2026) Nature Reviews Psychology.

The theory of sensorimotor association holds that the development of spatial perception does not arise solely from the independent maturation of sensory systems, but from the progressive alignment between sensory inputs and motor outputs, driven by the body’s action in space (see Figure 2). In the first months of life, the newborn already possesses rudimentary spatial codes in multiple modalities (vision, audition, touch), but these codes are initially separate, anchored to different reference frames (retinotopic for vision, somatotopic for touch, head-centered for hearing) and not yet aligned into a coherent multisensory representation (Bremner et al., 2008, 2013). The core computational problem for the developing brain is therefore to decide which senses belong to the same event and how to reconcile different spatial formats to form shared maps of the body and the surrounding environment. Beyond this bottom-up sensorimotor alignment, the emergence of intention marks a crucial transition. Early spontaneous and seemingly random movements provide the exploratory substrate for sensory-motor interactions (Garcia-Guzman et al., 2026), but around 3–6 months, these actions become increasingly organized around explicit goals, such as reaching for objects (Thelen et al., 1993). This shift from undirected motor activity to prospective, goal-directed action reflects the development of top-down control (Robson and Kuhlmeier, 2016), where children not only react to sensory input but actively anticipate and shape their sensory consequences (Corbetta and Fagard, 2017). Creative behaviors like drawing can thus be understood as an extension of these early skills into the cognitive domain, where intention transforms sensorimotor coordination into symbolic representation and externalized ideas (Elsner and Adam, 2021). In this framework, Gori et al. (2026) describe the development of spatial perception as the progressive solution of a cross-modal spatial binding problem, in which vision, when available, provides a privileged reference frame for building multisensory maps of external space. Vision offers a dense, instantaneous representation of the ambient layout “at a glance,” which can be used to calibrate noisier or more partial information from audition and touch. Developmental studies have shown that multisensory integration emerges gradually during the development (Bremner et al., 2013; Bruns and Roder, 2023; Gori et al., 2008; Nardini et al., 2008, 2010; Petrini et al., 2014), and that sometimes one modality serves as a privileged reference frame for calibrating other modalities (Gori et al., 2008, 2026). The theory of cross-modal calibration (Gori et al., 2008, 2026) proposes that, during development, the most accurate system for a given task, typically vision for the localization of objects in space, is used as a standard to realign the other systems, thereby reducing systematic biases and discrepancies across modalities, in agreement with other studies (Bruns and Roder, 2023). This calibration process, however, requires active sensorimotor experience, in which the child spontaneously links its own gesture to the perceptual changes it produces, initially through random, exploratory movements that evolve into goal-directed actions driven by intention (Garcia-Guzman et al., 2026; Thelen et al., 1993). As Rosander and von Hofsten (2004) illustrate, this transition from undirected motor activity to prospective, goal-oriented behavior around 12 weeks enables more efficient sensory motor association, transforming basic motor behaviors into sensorimotor representations of space.

A crucial role in this process is played by the emergence of visually guided reaching and grasping. Around 4–6 months, infants begin to reach systematically for visible objects, constructing stable associations between the retinal position of the target, the arm posture, the tactile and proprioceptive feedback of contact, and the outcome of the action. Each grasping attempt constitutes a micro-experience of sensorimotor learning: the brain must predict where the hand will be in space given a motor command, compare this prediction with the actual visual and tactile feedback, and update the spatial maps to reduce the error. In Bayesian terms, the system gradually builds a form of body schema that links internal body states, motor commands, and motor and sensory consequences, refining the estimation of hand and object positions in peripersonal space. Work by Bremner et al. (2008) shows that already in the first months infants are sensitive to visuo-tactile colocalization on the body: they prefer stimuli that touch the same body part where they see the contact occur, suggesting early associations between tactile and visual maps. Yet, the true sensorimotor reorganization occurs when the child begins to use the body actively to explore space, with visually guided reaching restructuring the links between somatotopic and external coordinates. Gori et al. (2026) emphasize that the development of spatial representations is intimately tied to bodily growth and the expansion of the child’s action repertoire. Rapid changes in body proportions (limb length, interocular distance) and the acquisition of new motor skills (crawling, creeping, walking) continuously alter the geometric relationship between senses and environment, imposing on the system a need for constant sensorimotor calibration. Sensorimotor association is therefore not a mere mechanism of “repeated association” between stimuli, but a dynamic process of alignment between sensory maps and action schemes that is updated across development. In this sense, space is coded (represented) by actions. In sum, from the perspective of sensorimotor association, the development of grasping and of multisensory spatial perception results from a continuous action-feedback-update loop, in which the body acts as a bridge between heterogeneous sensory systems. Reaching, object manipulation, and early attempts at grasping mark key moments when the child learns to map coherently what it sees, feels, and touches with its own motor intentions and action execution, laying the neural foundation for all subsequent forms of interaction with the world. Drawing as a natural training device for sensorimotor association and spatial calibration. If one adopts this same framework, children’s drawing can be reinterpreted as a powerful natural laboratory of sensorimotor association supporting intentional actions, in which the child repeatedly and systematically trains the links between perception and action, calibrating visual and bodily space. Unlike many everyday actions, graphic gestures leave a stable, tangible trace on paper, allowing the child to “see” the error, compare intention with outcome, and progressively adapt the loop between motor intention, execution of actions and perceived result. Each stroke functions as a learning trial in which the child formulates a sensorimotor prediction (“if I move my hand like this, the mark will go there”), receives immediate feedback on the position and shape of the trace, and updates its internal models of how arm and hand movements translate into a visual and tactile pattern.

**Figure 3:**
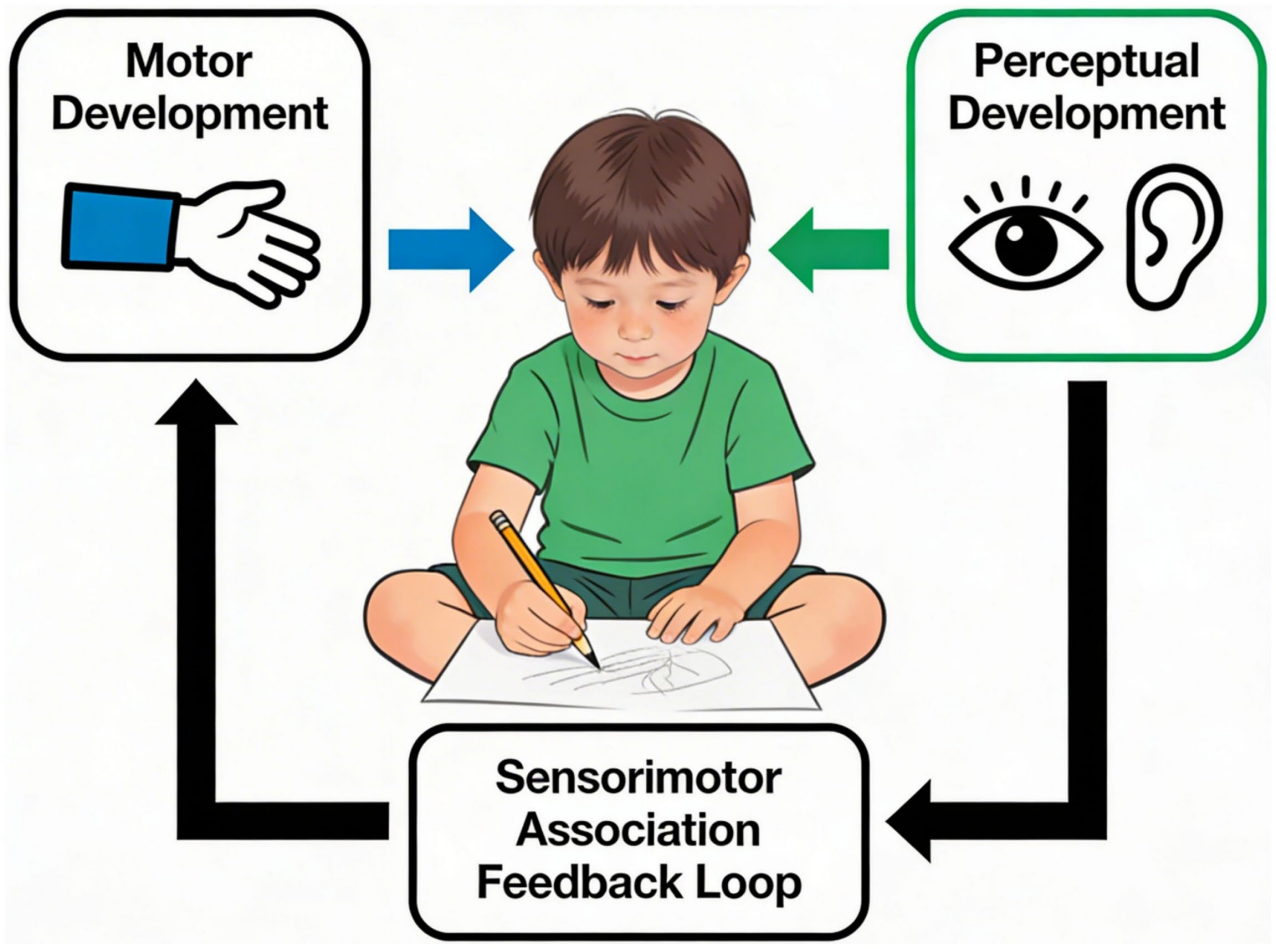
Sensorimotor feedback loop in children’s drawing. The diagram illustrates the bidirectional association between motor development (action via hand and arm) and perceptual development (visual and auditory input via eye and ear). Drawing acts as a natural training device where intentional motor actions produce immediate multisensory feedback (visual trace, tactile resistance, auditory cues), reinforcing the sensorimotor association and calibrating body-space maps.

At the multisensory level, drawing simultaneously engages vision, touch, proprioception, and often audition, creating an ecologically ideal context for the integration of the senses. The eye follows the emerging trace, the hand feels the resistance of the surface and the flow of the tool, the proprioceptive system encodes joint angles and trajectories, and the sound of the pencil or marker temporally structures the action. This spatio-temporal colocalization of multiple signals on the same object (the sheet) and the same effector (the hand) provides the brain with the very type of redundant evidence necessary to solve the cross-modal binding problem and calibrate sensorimotor maps. In other words, drawing places the child in a position to experience, in a controlled way, the relationship between where the hand is felt to be, where the mark is seen, and how the arm moved, facilitating the alignment of these codes. Compared with other forms of exploration, drawing has a unique property: visual feedback is not only transient, but it sediments over time, enabling a form of off-line learning. The child can return to its own scribble, recognize recurring patterns, compare its production with others and with the mental model of the object it intends to represent, thereby developing an increasingly explicit awareness of the link between gesture and form. This makes drawing a privileged context not only for the implicit calibration of sensorimotor maps, but also for the emergence of metacognitive abilities regarding the control of movement and the structure of graphic space (above/below, right/left, inside/outside the sheet). From the perspective of sensorimotor calibration, it is possible to hypothesize that the graphic stages described in the developmental literature (Kellogg, 1969) correspond to successive levels of refinement in visuo-motor control and calibration of spatial representation. In uncontrolled scribbling, the child mainly explores the basic gesture–mark causality, with large, poorly controlled movements that reflect a coarse, still immature sensorimotor map dominated by unisensory tactile and proprioceptive feedback. In controlled scribbling and the preschematic phase, the entry of a more stable visual guidance and the improvement of fine motor control allow the child to anchor the mark to specific spatial targets on the sheet, marking a first major step toward visuo-motor calibration. In the schematic and realistic phases, multisensory integration becomes more efficient and the child can use drawing to organize coherently proportions, perspective, and spatial relationships, signaling that sensorimotor maps are now sufficiently aligned to support complex representations. Drawing can therefore be thought of as a natural accelerator of cross-modal calibration: each drawing session offers numerous closely spaced opportunities to associate the final visual pattern with the motor history that produced it and the intended graphics, progressively reducing the discrepancy between the desired trajectory and the actual trajectory obtained. Drawing’s power as a calibration process between eyes, head and hand supports the hypothesis that a representation of current gaze direction may serve as a reference signal for arm motor control (Flanders et al., 1999). This echoes our pioneering robotics (Sandini et al., 1997) that proposed “motor-motor coordination,” mapping head-eye primitives to arm movements for grasping, implemented on BabyBot (Metta et al., 1999) and scaled to autonomous 3D reaching in iCub (Nori et al., 2007). Similarly, in drawing, fixating a point on the sheet guides the hand’s trace, training the same eye-head-to-limb alignment infants refine through grasping. A particularly relevant aspect of graphic practice is its iterative and self-reinforcing nature. Children tend spontaneously to repeat graphic patterns that yield “satisfying” outcomes, both esthetically and in terms of perceived motor control, while abandoning stroke forms that are tiring or unpredictable. This trial-and-error selection implements, at the behavioral level, an optimization process similar to the models of active inference described for the adult artist, in which the painterly gesture is continuously adjusted to minimize the discrepancy between the internal image and the sensory feedback. In this sense, children’s drawing can be viewed as the evolutionary prototype of pictorial making: a scaled-down cycle of active inference in which the child uses its body to sample space and refine its multisensory predictions. Active inference has been proposed as a useful framework for understanding action and perception within a single inferential architecture (Friston et al., 2010), in which behavior is shaped by the continual comparison between predicted and observed sensory consequences (Pezzulo et al., 2024). In the motor domain, related work has suggested that this framework may help account for goal-directed reaching, active sensing, multisensory conflict, and the coupling between decision-making and motor control, while also emphasizing that these accounts remain under active development (Priorelli et al., 2023a, b). More broadly, recent reviews have noted both the promise of active inference and the need for further empirical validation across different cognitive and neural domains (Hodson et al., 2024). Within this context, we suggest that children’s drawing may be considered a developmentally relevant case in which stroke production allows repeated sensorimotor sampling of action consequences, with each mark providing feedback that can support gradual refinement of internal models of body-space relations (Gori and Sandini, 2025). From this perspective, drawing may be viewed not only as an outcome of perceptual-motor development, but also as a practice through which sensorimotor contingencies are progressively calibrated during childhood (Gori et al., 2026). Moreover, drawing is likely the only everyday activity in which the child generates a spatially rich output from micro-variations of fine gesture, with an almost one-to-one correspondence between kinematic parameters (speed, curvature, amplitude) and the visual properties of the product (line thickness, direction, shape). The motivational background of children’s scribbling and drawing may therefore also be considered from the perspective of active inference, according to which agents can be guided not only by expected outcomes but also by the informational value of actions. From this view, drawing may be intrinsically motivating because it provides immediate, stable, and highly informative sensory consequences, allowing children to explore action-outcome relations while progressively reducing uncertainty about their own sensorimotor capabilities. Empirical work has shown that children adapt drawing behavior to their own motor variability and to the motivational context, suggesting that graphic action is sensitive to both intrinsic and extrinsic factors (Mohd Shukri and Howes, 2019). Thus, drawing may be understood as an activity that is both expressive and exploratory, where children may preferentially repeat actions that are informative, controllable, and perceptually rewarding. This makes drawing an exceptionally transparent context for the nervous system: small changes in control (e.g., slowing the hand, changing wrist angle) produce immediately visible differences in the mark, providing high-resolution feedback on the relationship between movement and outcome (Figure 3).

## Limitations of the model and open questions

The idea of drawing as a natural training device for sensorimotor association carries important theoretical and applied implications. Theoretically, it reverses the traditional view of drawing as a mere “output” of perceptual-motor development, instead proposing it as one of the drivers of multisensory calibration that enables “bottom-up” abilities such as precise grasping and fine manipulation as well as top-down, cognitive abilities such as mental simulation and active inference. From an applied perspective, if drawing effectively trains the alignment between sensory and motor maps, then educational and rehabilitative protocols that enhance multisensory graphic experience could be used to support spatial development in at-risk children, including those with visual deficits or motor coordination disorders. In this framework, the sheet becomes not only the symbolic space of representation, but a genuine sensorimotor calibration device through which the child learns to see, feel, and act in space in increasingly integrated and intentional ways. Despite the coherence of the proposed framework linking children’s drawing, multisensoriality, and sensorimotor association, several empirical and conceptual limitations must be explicitly acknowledged.

First, most of the evidence supporting drawing’s role as multisensory training comes from cross-sectional studies of motor development, multisensory integration, and graphic stages, rather than longitudinal studies tracking the same children over time to establish strong causal relationships. There is almost no literature that experimentally manipulates, in a controlled manner, the quantity and quality of graphic experience and measures its impact on standardized tasks of visuo-tactile, audio-tactile calibration, or body representation, as is common for other perceptual-motor training protocols.

A second limitation concerns the often-indirect nature of available measures: the hypothesis that drawing “calibrates” visual and bodily space rests largely on correlations between graphic complexity and the maturation of multisensory and motor abilities, but these measures have rarely been acquired in parallel with high-resolution tools (motion capture, eye-tracking, neuroimaging) in naturalistic graphic play contexts. This makes it difficult to discriminate whether drawing acts as a true driver of calibration or primarily reflects development driven by other sensorimotor experiences (reaching, locomotion, object manipulation).

A third critical point involves cultural generalizability. Most data on children’s drawing and graphic stages come from Western, school-structured contexts where paper and drawing tools are available and valued; the model implicitly assumes drawing as a universal experience, whereas in many cultures exposure to formal graphic practices is later, intermittent, or mediated by digital media. We do not know whether—and to what extent—the potential “natural training” offered by analog drawing can be substituted by digital equivalents. Finally, the process has been elaborated primarily for typical development: implications for atypical populations (motor coordination disorders, neurodivergences, early visual deficits) remain to be studied.

Overall, the idea of drawing as an embodied laboratory for sensorimotor calibration thus remains a theoretical framework requiring targeted research programs for confirmation, refinement, or potential revision.

## Conclusion

In this perspective, we propose that children’s drawing can be understood as a genuine evolutionary laboratory in which the prerequisites for adult painting are formed. The graphic gesture may constitute a privileged testing ground for the child, where it learns to solve, in miniature, the same problems of spatial, temporal, and sensorimotor binding that the adult brain must address in perception. Sensorimotor association, which in early grasping coordinates tactile, visual, and proprioceptive maps, is uniquely trained through drawing: each stroke leaves a stable trace that transparently links the kinematic parameters of the gesture and the contingent sensory feedback to the final result. In this sense, if adult painting embodies a process of active Bayesian inference on the environment, children’s drawing represents its original gymnasium, a natural training that calibrates space, body, and senses, transforming graphic play into a silent engine of perceptual, motor, and creative development.

Understanding drawing as sensorimotor training implies mapping motor development onto perceptual-multisensory and graphic development. Since the graphic gesture provides the child with immediate feedback on the relationship between movement and sensory consequence, the emergence of drawing stages should proceed in parallel with motor milestones (from proximal to distal control, from palmar to tripod grip) and the progressive optimization of multisensory integration. Current evidence documents strong parallels between motor maturation, perceptual-spatial abilities, and graphic complexity, but does not clarify whether drawing is merely an effect of these processes or actively contributes to accelerating them. If it acts as a multisensory trainer, a controlled increase in graphic experience should produce measurable changes in visuo-tactile calibration tasks, body representation, and fine motor skills, beyond the effects of mere chronological development or other motor experiences.

In this work, literature on children’s drawing and the neural correlates of adult drawing shows that the graphic act engages networks dedicated to visuo-motor transformation and fine gesture control, suggesting a structural continuity between graphic production and action control systems. In the adult domain, studies on perceptual training indicate that brief periods of visual training with feedback can modify the audiovisual integration window and the relative weighting of senses, demonstrating the plasticity of experience-driven multisensory integration. Approaches from art therapy and sensorimotor art therapy further support that guided and bilateral drawing can strengthen motor schemes and body-emotional regulation. Similarly, data on drawing development document tight links between fine motor skills, perceptual-spatial abilities, and graphic complexity, suggesting at least strong covariation across these trajectories. Yet, drawing has been treated predominantly as an outcome of development, rather than a variable capable of modifying it.

To conclude, our theoretical proposal hypothesizes that drawing offers the child a unique context in which every motor command generates informative visual, tactile, and proprioceptive feedback, producing progressive refinement of sensorimotor integration models. Formulated in Bayesian terms, this idea considers children’s drawing as a sequence of active experiments with visible outcomes, through which the nervous system updates its generative models of the association between motor states and sensory consequences. Each stroke is a predictive sample: the child anticipates the line’s direction, observes the deviation, and reduces error across successive iterations, improving control and mapping between bodily coordinates and sheet coordinates. This perspective thus aims to pave the way for longitudinal studies and targeted experimental interventions to test its neurobehavioral and educational implications.

## Data Availability

The original contributions presented in the study are included in the article/supplementary material, further inquiries can be directed to the corresponding author.
